# Targeting inflammation for the treatment of Diabetic Kidney Disease: a five-compartment mechanistic model

**DOI:** 10.1186/s12882-022-02794-8

**Published:** 2022-06-13

**Authors:** Alexis Hofherr, Julie Williams, Li-Ming Gan, Magnus Söderberg, Pernille B. L. Hansen, Kevin J. Woollard

**Affiliations:** 1 Research and Early Clinical Development, Cardiovascular, Renal and Metabolism, AstraZeneca, BioPharmaceuticals R&D, Gothenburg, Sweden; 2 Renal Division, Department of Medicine, Medical Center, Faculty of Medicine, University of Freiburg, Freiburg, Germany; 3 Bioscience Renal, Research and Early Development, Cardiovascular, Renal and Metabolic, AstraZeneca, BioPharmaceuticals R&D, Gothenburg, UK; 4 Department of Molecular and Clinical Medicine, Department of Cardiology, Sahlgrenska Academy, University of Gothenburg, Sahlgrenska University Hospital, Gothenburg, Sweden; 5 Cardiovascular, Renal and Metabolic Safety, Clinical Pharmacology and Safety Sciences, AstraZeneca, BioPharmaceuticals R&D, Gothenburg, Sweden; 6 Wallenberg Center for Molecular and Translational Medicine, Institute of Neuroscience and Physiology, the Sahlgrenska Academy, University of Gothenburg, Gothenburg, Sweden; 7 Centre for Inflammatory Disease, Imperial College London, London, UK

**Keywords:** Diabetes, Diabetic kidney disease, Inflammation, Biomarkers

## Abstract

Diabetic kidney disease (DKD) is the leading cause of kidney failure worldwide. Mortality and morbidity associated with DKD are increasing with the global prevalence of type 2 diabetes. Chronic, sub-clinical, non-resolving inflammation contributes to the pathophysiology of renal and cardiovascular disease associated with diabetes. Inflammatory biomarkers correlate with poor renal outcomes and mortality in patients with DKD. Targeting chronic inflammation may therefore offer a route to novel therapeutics for DKD. However, the DKD patient population is highly heterogeneous, with varying etiology, presentation and disease progression. This heterogeneity is a challenge for clinical trials of novel anti-inflammatory therapies. Here, we present a conceptual model of how chronic inflammation affects kidney function in five compartments: immune cell recruitment and activation; filtration; resorption and secretion; extracellular matrix regulation; and perfusion. We believe that the rigorous alignment of pathophysiological insights, appropriate animal models and pathology-specific biomarkers may facilitate a mechanism-based shift from recruiting ‘all comers’ with DKD to stratification of patients based on the principal compartments of inflammatory disease activity.

## Background

Risk factors, such as genetic predisposition, sedentary lifestyle, overweight and unhealthy diet, have resulted in an unprecedented prevalence of type 2 diabetes [1]. Consequently, kidney disease secondary to diabetes (diabetic kidney disease, DKD) has become the leading cause of kidney failure, with more than 400 000 deaths among adults worldwide in 2017 [2, 3]. However, clinical presentation and end organ damage vary widely among patients with type 2 diabetes, with around one in three patients developing DKD with albuminuria > 300 mg/day and/or glomerular filtration rate (GFR) < 60 mL/min/1.73 m^2^ [3].

The identification of molecular pathways connecting systemic and local inflammation to the pathology of type 2 diabetes and DKD has sparked growing interest in targeting inflammation to prevent disease progression, as well as improving patient risk stratification by inflammatory biomarkers. Sub-clinical chronic inflammation with multi-organ crosstalk is increasingly recognized as a driver of linked cardiovascular, renal and metabolic disease states [4–7].

Overt immune cell infiltration was not historically considered as one of the classical histopathological signs of DKD: glomerular sclerosis and mesangial expansion, first noted in the 1930s, accompanied by thickening of glomerular and tubular basement membranes, podocyte injury and tubulointerstitial fibrosis [8–10]. However, inflammatory biomarkers correlate with mortality and end-stage renal disease in patients with DKD [5]. Kidney failure, furthermore, causes systemic inflammation that contribute to morbidity and mortality among patients with chronic kidney disease [6]. Only since the discovery of macrophage infiltration as a key histopathological feature in the 1990s has it become recognized that fibrosis and sclerosis in the diabetic kidney are part of a chronic inflammatory disease process that correlates with disease progression [11–15]. Inflammation may therefore represent a key factor in development of DKD for a substantial subgroup of patients with type 2 diabetes.

**Fig. 1:**
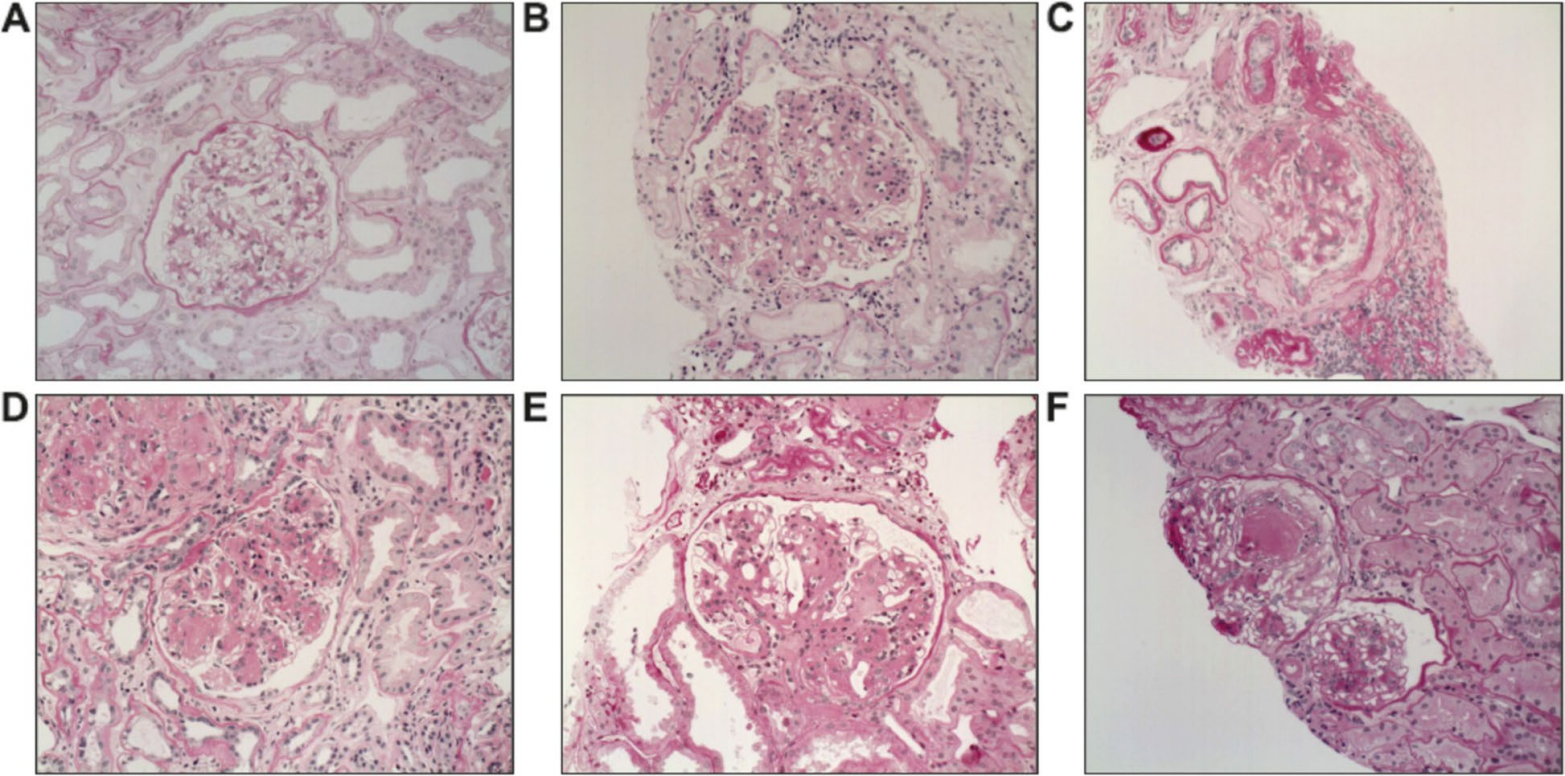
Histology showing the complex and heterogeneous glomerular pathology in DKD. **A**: Minimal to mild glomerular pathology with mild mesangial expansion; Tervaert class I–IIa [20]. **B**: Severe mesangial expansion and hypercellularity; Tervaert class IIb. **C**: Ischaemic phenotype with collapse of glomerular segments, segmental sclerosis and mild mesangial expansion; Tervaert class IIa. **D**: Severe mesangial expansion, Kimmelstiel-Wilson nodule without mesangiolysis; Tervaert class III. **E**: Hyperfiltrating phenotype with enlarged glomerular tuft, perihilar capsular adhesion and severe mesangial expansion; Tervaert class IIb. **F**: Mild mesangial expansion, Kimmelstiel-Wilson nodule with mesangiolysis; Tervaert class III

The complex and heterogeneous pathophysiology of DKD presents serious challenges to the development of effective treatments [16, 17]. Chronic kidney disease in a patient with type 2 diabetes may be a direct result of diabetes, exacerbated by diabetes or unrelated to diabetes [18, 19]. At present, these disease states can only be differentiated by histological analysis of kidney biopsies [18, 19]. Although classifications of DKD have been proposed [20], the lack of consistent use of biopsies as a diagnostic tool in diabetic patients with proteinuria calls into question the general translatability of observations in cohorts of patients who have undergone biopsy to the general diabetic population. Examples of the diverse histopathology of DKD are shown in Fig. 1.

Multiple molecular pathways contribute to the chronic, sub-clinical, non-resolving inflammation that characterizes DKD in many patients (Table 1) [21–23]. Kidney cell injury or stress leads to release of damage-associated molecular patterns that activate pro-inflammatory intracellular signaling pathways [24]. Noxious biochemical stimuli resulting from high plasma glucose and lipid levels include oxidative stress, reactive oxygen species, glycated proteins, and oxidized lipids [23, 25]. In addition, glycated proteins can directly activate the complement system and initiate pro-inflammatory signaling [21–23]. High capillary blood pressure places potentially damaging high shear forces on cells, and these are exacerbated by stiffness due to fibrosis [26]. In response to ongoing activation of innate immune damage sensors, kidney endothelial cells, mesangial cells and podocytes produce multiple inflammatory cytokines, chemokines and adhesion molecules. These activate and recruit monocytes and macrophages, leading to further cascading inflammatory responses [23, 27]. The ongoing chronic inflammation results in extracellular matrix deposition and fibrosis, driven both by kidney-resident cells and by recruited cells of the innate immune system [28].

**Table 1:** Examples of inflammatory molecular pathways in diabetic kidney disease

Damage-associated molecular patterns	HMGB1, HSPs, fibronectin Advanced glycation end-products IL-33
Pattern-recognition molecules	TLR2, TLR4 NLR RAGE MBL (complement)
Intracellular signaling	JAK/STAT, NF-kB, Nrf2 NLRP3 inflammasome
Chemokines	CCL2, CCL5, CSF1, CXCL1, CXCL16, CXCL-10, CXCL-16, IL-8
Cytokines	IL-6, TNF-α, IL-1β, IL-18, IL-17A, TGF-β1, CX3CL1
Adhesion molecules	ICAM-1, VCAM-1, Galectin-3, Integrin αVβ3, LFA-1, VAP-1
Pro-fibrotic mediators	PDGF, TGF-β

Based on Donate-Correra et al., 2020 [21]; Rayego-Mateos et al*.,* 2020 [22]; Tang et al*.,* 2020 [23]; Vallon et al*.,* 2020 [29]; and Scurt et al*.*, 2019 [30]

Abbreviations: *CCL* C–C motif ligand, *CSF1* colony stimulating factor 1, *CXCL* chemokine (C-X-C motif) ligand, *HMGB1* high mobility group box 1, *HSP* heat shock protein, *ICAM-1* intracellular adhesion molecule, *IL* interleukin, *JAK* Janus kinase, *LFA-1* lymphocyte function-associated antigen 1, *MBL* mannose-binding lectin, *NLR* nucleotide-binding oligomerization domain-like receptor, *NLRP3* NACHT LRR and PYD domains-containing protein 3, *PDGF* platelet-derived growth factor, *RAGE* receptor for advanced glycation end-products, *STAT* signal transducer and activator of transcription, *TGF-β*, transforming growth factor β, *TLR* toll-like receptor, *TNF-α* tumor necrosis factor α, *VAP-1* vascular adhesion protein 1, *VCAM-1* vascular cell adhesion molecule

Several biomarker studies indicate that inflammation predicts and precedes development of albuminuria in patients with type 2 diabetes or DKD [30–35]. Still, clinical trials of novel anti-inflammatory therapies in patients with DKD have not demonstrated consistent benefit on renal outcomes, despite improvements in biomarker outcomes (as reviewed in detail below) [36–38]. The renal side effects of non-steroidal anti-inflammatory drugs, for example, preclude their use in patients with DKD, and may stem partly from their effects on renal prostaglandin signaling [39]. A better understanding of how a chronic inflammatory microenvironment drives the development and progression of DKD may unlock the potential of anti-inflammatory therapy, by allowing segmentation of patients according to their specific inflammatory activity.

## Five-compartment model of diabetic kidney disease immunopathology

**Fig. 2:**
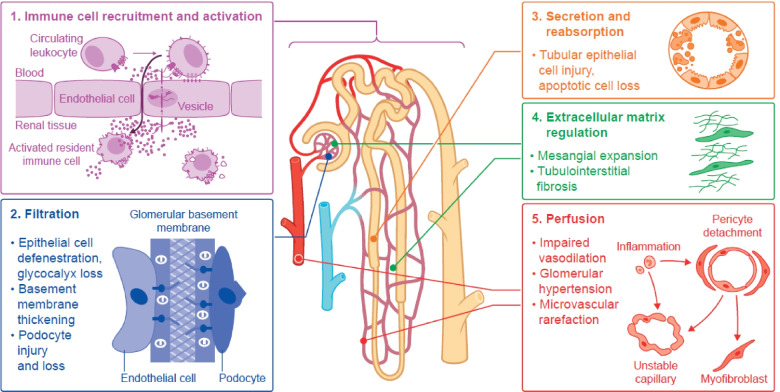
Five-compartment model of impairment due to chronic inflammation in diabetic kidney disease. Conceptual model of five functional and structural compartments that can be affected by immunopathology in patients with diabetic kidney disease. The model provides a framework for linking pre-clinical models of disease pathways with predominant immunopathology in particular patient types. Stratification of patients based on predominant immunopathology in the five-compartment model may enable targeting of the right treatments to the right patients

To conceptualize the complex immunopathology of the kidney during diabetes, we highlight five key compartments of kidney function and structure that are impaired by chronic inflammatory disease activity (Fig. 2). The five compartments are not mutually exclusive, and their relative importance may vary among patients. We show how the model can help to align pre-clinical models for target validation with clinical efficacy endpoints and patient selection criteria. Stratifying patients with DKD based on their predominant immunopathology could enable testing of novel anti-inflammatory drugs in the patients most likely to benefit. As discussed in the section on clinical trial design, this approach would currently require kidney biopsy, but may in future be based on circulatory, urinary or imaging biomarkers [34, 40, 41].

### Immune cell recruitment and activation

During stress or inflammation, such as those triggered by hypoxia, ATP or other damage-associated molecular patterns (DAMPs) in the DKD microenvironment, can result in kidney cells expressing chemokines and adhesion molecules. Together this attracts circulating leukocytes into the damaged renal tissue and activates resident cells [23, 42].

Glomerular endothelial cells, mesangial cells, podocytes and tubular epithelial cells express multiple cytokines, including the interleukin (IL) family members (e.g. IL-1, IL-6 and IL-19) tumor necrosis factor-α (TNF-α); multiple chemokines, including C–C motif ligand 2 (CCL2); and multiple adhesion molecules, including vascular cell adhesion molecule 1 (VCAM-1), intercellular adhesion molecule 1 (ICAM-1) and selectins (Table 1) [27]. Activated monocytes/macrophages recruited into the kidney amplify the response by producing pro-inflammatory cytokines (e.g. IL-1β, TNF-α, IL-6 and IL-18) [22]. Activated immune cells also produce multiple molecules that can cause further renal injury, including metalloproteases, reactive oxygen species, advanced glycation end-products and complement proteins [22, 43, 44].

Recruitment of monocytes and macrophages into the kidney is a key step in the pathophysiology of DKD [22]. Macrophage accumulation in the kidney correlates strongly with serum creatinine levels, interstitial myofibroblast accumulation and interstitial fibrosis scores [12, 14, 15]. Resident macrophages and dendritic cells in the tubulointerstitium also contribute to disease progression by recruiting and activating lymphocytes [45]. Non-classical renal ‘patrolling’ monocytes may further orchestrate immune cell responses at the glomerular vascular interface, including recruitment and activation of neutrophils [46, 47]. Interestingly, cell-to-cell communication of resident immune cells, such as macrophages, with renal cells has also been shown to regulate transendothelial transport of immune complexes, as well as other immunoregulatory pathways [23, 48].

Macrophages may progress from the M1-like pro-inflammatory phase to the M2-like tissue repair stage, however, both forms coexist during chronic inflammation in DKD and represent a spectrum of macrophage phenotypes, leading to fibrosis [21]. Pro-fibrotic mediators released by macrophages induce extracellular matrix deposition, leading to fibrosis and impaired renal function [10, 49].

### Filtration

In the early stages of DKD, glomerular hyperfiltration may cause kidney injury and contribute to disease progression by increasing physical stress and oxygen demand to drive reabsorption [29, 50]. Current standard of care, inhibition of the renin angiotensin aldosterone system and sodium-glucose co-transporter-2 (SGLT2), is reducing glomerular injury and can provide significant clinical benefit for patients [29, 50, 51]. However, isolated glomerular hyperfiltration does not predict development of advanced DKD, consistent with a pathophysiological role for chronic inflammation [51].

**Fig. 3:**
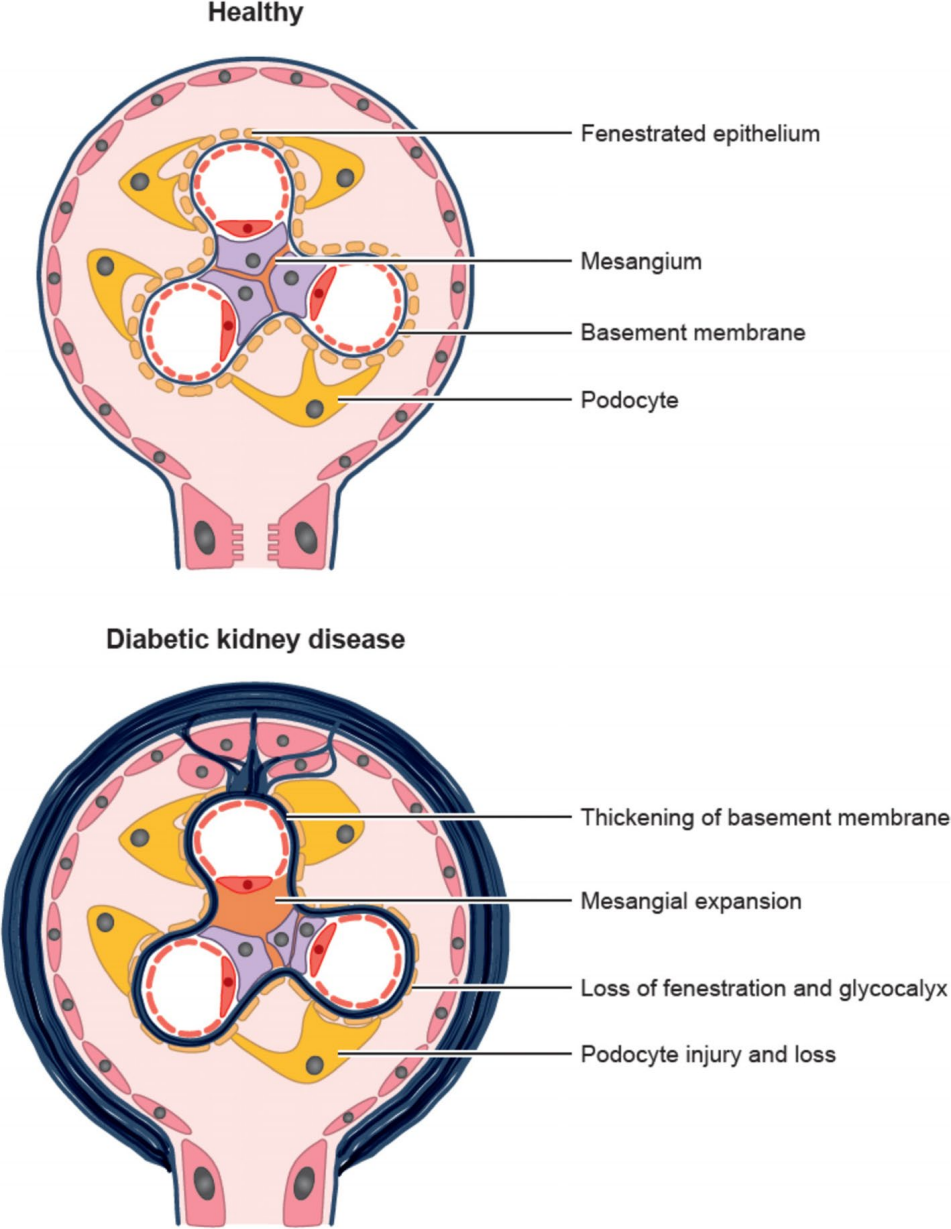
In DKD chronic inflammation adversely affects all three components of the glomerular filter: the fenestrated endothelium, the basement membrane and the epithelium, comprising podocytes and slit diaphragms

Chronic inflammation adversely affects all three components of the glomerular filter: the fenestrated endothelium, the basement membrane and the epithelium, comprising podocytes and slit diaphragms (Fig. 3) [52]. TNF-α secreted by resident and infiltrating macrophages is cytotoxic to glomerular mesangial and epithelial cells, and impairs glomerular hemodynamics and filtration [27]. ICAM-1 and E-selectin expression in the glomerular endothelium is induced by pro-inflammatory cytokines and promotes leukocyte recruitment [21, 27].

**Fig. 4:**
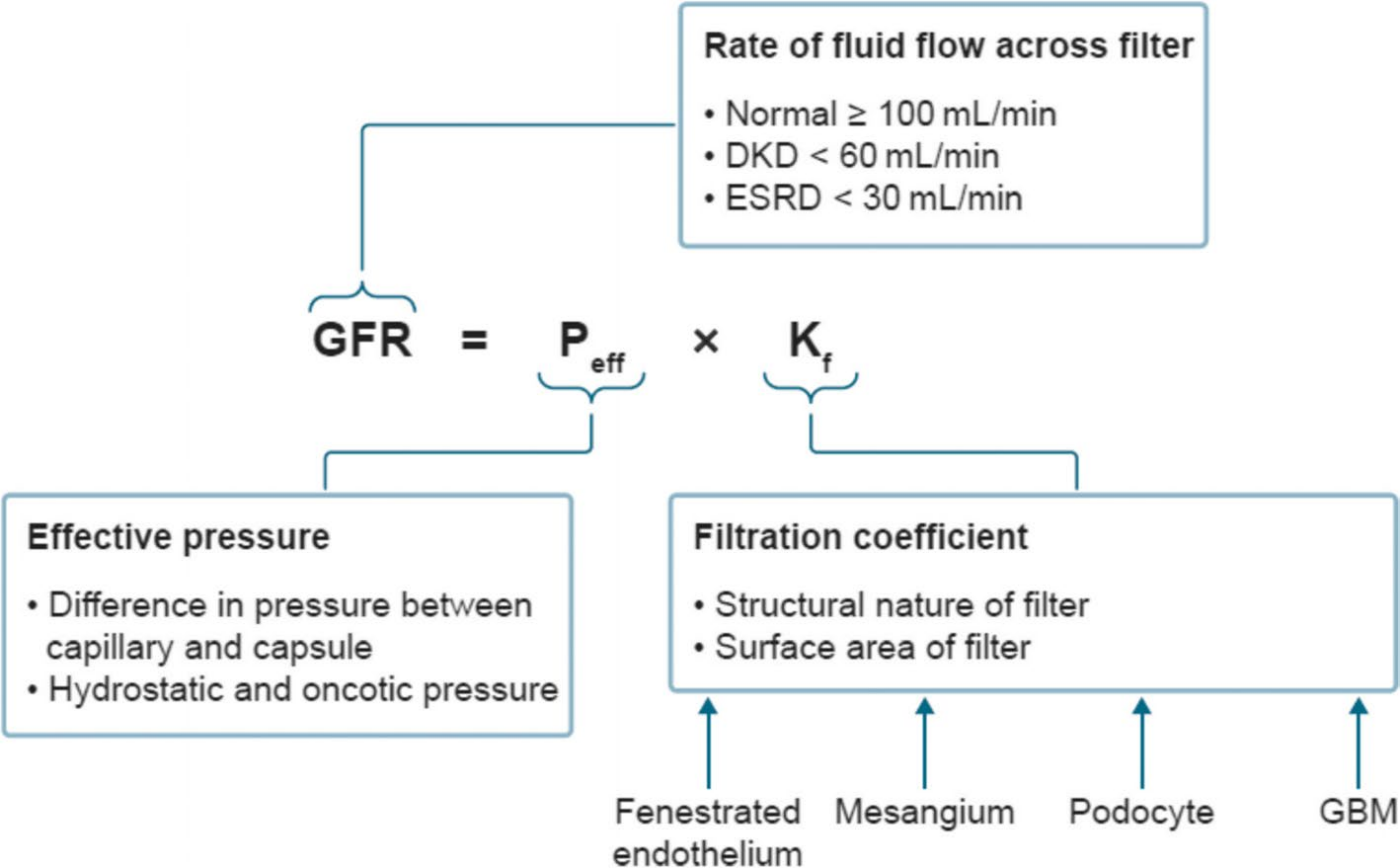
Determinants of glomerular filtration rate. Classic nephrology equation describing how GFR depends on the pressure across the filter and its coefficient of filtration, which is determined by its structural composition and surface area. In DKD, structural composition is impaired by loss of endothelial fenestration, injury and loss of podocytes and thickening of the glomerular basement membrane, and surface area is reduced by mesangial expansion due to fibrosis. *ESRD* End stage renal disease

Inflammation-mediated alterations impair the filtration function of the glomerulus, which relies on size and charge separation. Filtration rate depends on the pressure across the filter and its coefficient of filtration, which is determined by its structural composition and surface area (Fig. 4). The filter’s size and charge selectivity are lost and its permeability increases as glomerular endothelial cells lining the capillaries lose their characteristic fenestrations and as their overlying glycocalyx layer is degraded [53]. Thickening of the glomerular basement membrane due to extracellular matrix deposition correlates with progression of albuminuria and declining GFR, and is one of the earliest histological signs of DKD [8, 52]. The membrane may double in thickness in people with diabetes, from a normal thickness of around 300–350 nm [8]. Podocyte stress, injury and eventual loss are also key factors in disease progression. Podocytes create slit diaphragms for filtration with their foot processes, secrete basement membrane components, communicate with fenestrated endothelial cells, and endocytose proteins that pass through the barrier [54]. Injured podocytes retract their foot processes, disrupting the structure of the filter, and this ‘effacement’ leads to development of proteinuria [55].

### Resorption and secretion

Excess protein, advanced glycation end-products, growth factors and complement proteins in glomerular filtrate harm proximal tubular epithelial cells and activate pro-inflammatory responses [45]. Inflammatory and pro-fibrotic cytokines trigger de-differentiation of tubular epithelial cells, leading to loss of resorptive and secretory activities and acquisition of mesenchymal cell-like features, including production of extracellular matrix [56]. Disruption of normal uptake of proteins from the glomerular filtrate by proximal tubular epithelial cells contributes to development of proteinuria [57]. The relative contribution of tubular pathology might increase if SGLT2 inhibitor use slows progression of glomerular pathology, potentially increasing the importance of this compartment. Tubular epithelial cells produce pro-inflammatory and pro-fibrotic cytokines and chemokines including CCL2, IL-8, TGF-β and CCL5 (also known as RANTES) [29]. The resulting inflammation leads to tissue damage, with resulting hypoxia, apoptosis, tubular atrophy, disconnection of the tubule from the glomerulus, and eventual renal failure [58].

### Extracellular matrix regulation

Mesangial expansion with eventual fibrosis is a histopathological hallmark of DKD. Excessive accumulation of extracellular matrix leads to fibrosis and impaired function throughout the chronically inflamed diabetic kidney [59]. Fibrosis prevents mesangial cells from expanding and contracting to control capillary blood pressure and the surface area of the glomerular filter, with resulting impairment in filtration [60]. Tubulointerstitial fibrosis expands the space between the tubular basement membrane and the peritubular capillaries, leading to reduced blood flow, hypoxia, and further epithelial damage and inflammation, and eventual tubular atrophy [59].

Pro-fibrotic mediators released by activated macrophages and injured epithelial cells upregulate extracellular matrix production in various cell types [22, 61]. These include mesangial cells, which normally secrete extracellular matrix to provide structural support for the glomerular capillary tuft [60], and perivascular fibroblasts, which normally provide structural support to the kidney microvasculature [62]. Potential biomarkers of renal fibrosis include TGF-β and matrix metalloproteinase 2 (MMP-2) [63]. TGF-β produced by pro-fibrotic macrophages upregulates expression of extracellular matrix proteins in kidney endothelial and epithelial cells [59, 61]. Oxidative and mechanical stress also directly enhance extracellular matrix production and contribute to fibrosis [61].

### Perfusion

Ischaemia is a key trigger of inflammation in DKD, especially in the highly metabolically active tubular epithelium. Impaired perfusion leads to hypoxic injury, triggering inflammation, fibrosis, tubular atrophy and progression of DKD [64]. Type 2 diabetes is characterized by a more ischaemic kidney phenotype than the oxidative and proliferative pattern seen with type 1 diabetes [65, 66]. Oxygen demand in the tubules leads to sustained activation of the intrarenal renin–angiotensin–aldosterone system, causing glomerular capillary hypertension that further damages glomerular endothelial and mesangial cells and podocytes [67]. Excessive oxygen levels in glomerular tissues lead to oxidative stress and formation of reactive oxygen species and advanced glycation end-products, with resulting inflammation and fibrosis [64]. Angiotensin II also directly activates pro-inflammatory and pro-fibrotic signaling pathways, contributing to endothelial cell injury and loss [68].

High glucose levels and advanced glycation end-products impair nitric oxide production by kidney endothelial cells, leading to impaired vasodilation in patients with DKD. Nitric oxide deficiency exacerbates oxidative stress, leading to further dysfunction and injury not only of epithelial cells but also adjacent cells, including podocytes [69]. Under oxidative stress, dimeric endothelial nitric oxide synthase decouples and produces superoxide instead of nitric oxide, which exacerbates both oxidative stress and nitric oxide deficiency [70]. Microvascular rarefaction in DKD eventually results from damage and apoptotic loss of endothelial cells together with impaired function of endothelial progenitor cells due to the impact of oxidative stress, advanced glycation end-products, and an inflammatory, pro-fibrotic milieu [69].

## Pre-clinical research using the five-compartment model

The five-compartment model provides a framework for rationalizing the array of different animal models of type 2 diabetes, with the aim of linking pre-clinical research to clinical development based on pathophysiology in particular patient groups. In this section, we summarize the key features of commonly used pre-clinical models and how they translate to the five described compartments of inflammation. This may help with selection of the best pre-clinical model to use to define pathophysiological mechanisms in DKD. However, none of the available animal models faithfully replicates all aspects of DKD in humans, most notably because none involves progression to renal failure.

### Rodent models

Most rodent models of DKD involve induction of diabetes-like phenotypes by streptozotocin treatment, spontaneous mutations, or genetic manipulation in laboratory mice (*Mus musculus*). Current mouse models have been successfully used with clinical standard of care in DKD and may address some inflammatory compartments, but study of ‘immune cell recruitment’ and ‘perfusion’ have remained challenging (Table 2) [71].

**Table 2:** Mouse models of diabetic kidney disease

Strain	Model compartments (see Fig. 1)	Diabetogenic mechanism	Reported features
**Type 1 diabetes**			
C57BL/6	Immune cell recruitment, resorption, filtration	Streptozotocin	Mild glomerular and tubulointerstitial damage, mild albuminuria, GFR increase, hyperglycemia
Akita (*Ins2*^C96Y^) on C57BL/6	Immune cell recruitment, structural support, filtration	Toxic mutation in insulin 2 gene	GBM thickening, mesangial expansion, albuminuria, hyperglycemia, hypertension
* ApoE*^−/−^ on C57BL/6	Immune cell recruitment, resorption, filtration	Streptozotocin + hyperlipidemia	Glomerular and tubulointerstitial damage, albuminuria, hyperglycemia
* Nos3*^−/−^ on C57BL/6	Immune cell recruitment, filtration	STZ + NO deficiency	Glomerular fibrosis, albuminuria, hyperglycemia
BALB/c	Immune cell recruitment, filtration	Streptozotocin	Glomerular damage, hyperglycemia; no change in GFR
DBA/2 J	Immune cell recruitment, resorption, structural support	Streptozotocin	Glomerular fibrosis, tubulointerstitial damage, hyperglycemia
Akita (*Ins2*^C96Y^) on DBA/2 J	Immune cell recruitment, resorption, filtration, structural support	Toxic mutation in insulin 2 gene	Albuminuria, hyperglycemia
OVE on FVB	Immune cell recruitment, resorption, filtration, structural support	Calmodulin mutation and toxic protein accumulation	Glomerular and tubulointerstitial fibrosis, albuminuria, GFR reduction, hyperglycemia, hypertension
TTRhRen on FVB	Immune cell recruitment, resorption, structural support	Hypertension + streptozotocin	Tubulointerstitial fibrosis, mesangial expansion, albuminuria, GFR decrease, hyperglycemia, hypertension
CD1	Immune cell recruitment, resorption, structural support	Streptozotocin	Tubulointerstitial fibrosis, mesangial expansion, albuminuria, hyperglycemia
129/SV	Immune cell recruitment, filtration	Streptozotocin + 2 renin receptors	Albuminuria, hyperglycemia
Akita (*Ins2*^C96Y^) on 129/SV	Immune cell recruitment, structural support	Toxic mutation in insulin 2 gene	Mesangial expansion, albuminuria, hyperglycemia, hypertension
KKH1J	Immune cell recruitment, filtration	Streptozotocin	Glomerular damage, albuminuria, hyperglycemia
NOD Mice	Filtration, structural support	Genetic obesity + streptozotocin	Hyperglycemia
**Type 2 diabetes**			
* Db/db* on C57BL/Ks	Immune cell recruitment, structural support	Leptin resistance	Mesangial expansion, albuminuria, hyperglycemia
* Db/db Nos3*^−/−^ on C57BL/Ks	Immune cell recruitment, filtration, and structural support	Leptin resistance + NO deficiency	Albuminuria, GFR decrease, hyperglycemia
* Ob/ob*	Immune cell recruitment, filtration, and structural support	Leptin deficiency	Hyperglycemia
* Ob/ob* on BTBR	Immune cell recruitment, filtration, and structural support	Leptin deficiency + hyperinsulinemia	Hyperglycemia
KK and KKay	Immune cell recruitment, filtration, and structural support	Agouti gene	Albuminuria, hyperglycemia, hypertension

Based on Nguyen et al., 2019 [72]

Abbreviations: *GFR* glomerular filtration rate, *NOD* non-obese diabetic, *FVB* Friend leukaemia virus B, STZ + NO deficiency, streptozotocin + nitric oxide deficiency

Streptozotocin is a cytotoxic glucose analogue that ablates pancreatic islet β cells, with severity of diabetes-like features depending on the mouse strain. In C57BL/6 J or Balb/c mice, streptozotocin induces only mild or moderate disease, but severity can be increased using genetic modification, crossing with other strains or a high-fat diet. Combing streptozotocin with hyperlipidaemia by knocking out *Apoe* (encoding apolipoprotein E) accelerates and worsens renal injury in C57BL/6 mice [8, 72]. Mutations in Akita and OVE26 mice cause pancreatic β cells toxicity, and the severity of the diabetes-like disease also varies depending on mouse strain. In *db/db* mice, a genetic defect in the leptin receptor leads to obesity, diabetes, and some signs of DKD. Surgical removal of one kidney (uninephrectomy) accelerates progression of kidney pathology in *db/db* mice [72]. Genetic knockout of *Nos3* (encoding eNOS) or overactivation of the renin–angiotensin system in *TTRhRen* mice also accelerate loss of kidney function in mouse models (Table 2) [8, 72].

### Non-rodent models

Zebrafish (*Danio rerio*) have been used to study ‘filtration’ and ‘immune cell recruitment’ in DKD. Zebrafish offer low cost, high throughput, and an advanced transgenic toolbox for molecular genetic manipulation, at the cost of potential low translatability to human disease. The kidney spontaneously regenerates in fish, providing challenges to test the effectiveness of potential therapeutics. Zebrafish have been used to study genetic variants that lead to podocyte damage and DKD [73, 74].

Models in domestic pigs (*Sus scrofa*) recapitulate features of DKD in all five compartments. A high-fat high-fructose diet induces renal hypertension, endothelial dysfunction and inflammation, and streptozotocin plus high-fat diet induces renal injury and proteinuria. Genetic manipulation to insert the Akita mutation into pigs leads to diabetes, but changes in kidney function have not yet been described [72].

Non-human primates (*Macaca mulatta*) are the gold standard for animal models of human kidney disease, but ethical considerations, high cost and the difficulty of genetic manipulation limit respective investigations. Streptozotocin administration leads to histopathological changes, proteinuria and impaired GFR, with the fastest disease progression observed using uncontrolled blood glucose levels and a high-fat, high-salt diet [72]. Still, aged dysmetabolic non-human primate models may offer the most suitable disease model, particularly as aging may be an important factor in DKD [75].

### Organoids

Organoids are three-dimensional tissue structures derived by in vitro differentiation of induced pluripotent or other stem cells [76]. Blood vessel organoids with capillary networks, develop thickening of the basement membrane after exposure to high glucose levels and inflammatory cytokines, potentially providing a model for investigation of microvascular aspects of DKD [77]. The latest kidney organoids comprise connected nephrons and collecting ducts, and research is ongoing into nephropathy and fibrosis for DKD target validation [76].

## Clinical trial design using the five-compartment model

The recent addition of SGLT2 inhibitors to angiotensin-converting enzyme inhibitors (ACEi) and angiotensin receptor blockers (ARB) as standard of care for DKD promise a reduction in risk for adverse renal and cardiovascular outcomes in patients with type 2 diabetes, most likely via a hemodynamic and metabolic mechanism of action [78–81]. Nevertheless, many patients with DKD remain at high risk of kidney disease progression and still bear the majority of the increased risk of cardiovascular and all-cause mortality among patients with type 2 diabetes [82, 83]. This indicates a persistent need for novel treatments that target different pathophysiological pathways such as inflammation [21–23].

Diagnostic inaccuracy in DKD is a major challenge in part due to a classification system categorizing kidney disease according to chronicity and severity based on non-specific markers; and in part due to the unique, often multimorbid heterogeneity of patients with DKD [84]. Several important international efforts, including the U.S. Kidney Precision Medicine Project and the European BEAt-DKD Consortium, have been initiated to better characterize the pathology of human DKD and the factors involved in its progression ([85] https://www.beat-dkd.eu/). Meticulous alignment of patient’s needs, scientific hypothesis, pre-clinical model systems and clinical studies will be paramount to efficiently translate relevant findings into novel treatment paradigms [86]. The five-compartment model presented here aims to contribute to this endeavor by providing a function-based framework to map the diverse pathological mechanisms in renal inflammation onto central, measurable kidney functions.

In both type 1 and type 2 diabetes, rate of renal function decline and kidney failure are associated with circulating inflammatory proteins, including tumor necrosis factor receptors 1 (TNF-R1) and TNF-R2 [5, 35, 87–90]. Within the kidney, innate and adaptive immune responses have been correlated with structural lesions, including TLR4- and CCL2-based pathways [91]. Markers of inflammation may therefore be useful for both prognosis as well as treatment response in DKD [92]. However, the relationship between systemic and local low-grade inflammation and the glomerular, vascular or tubulointerstitial damage in patients remains rather unclear [93]. For example, infliximab (anti-TNF monoclonal antibody) and etanercept (TNF-R2-Fc) decreased albuminuria in animal models of diabetes and 24 weeks of 4 mg baricitinib (a JAK1/2 inhibitor; *n* = 25) significantly reduced morning urinary albumin-to-creatine ratio (UACR; –41%), as well as plasma TNF-R1 and TNF-R2, in a small phase 2 study in DKD relative to placebo (*n* = 27) [34, 94–96].

### Patient selection and endpoints in published and ongoing studies

No anti-inflammatory drugs for the treatment of DKD have progressed beyond phase 2 clinical trials, except maybe for finerenone (Table 3). Finerenone, a non-steroidal selective mineralocorticoid receptor antagonist that induces natriuresis with reduced hyperkalaemia compared with steroidal antagonists (e.g. spironolactone), may retain some potentially beneficial anti-inflammatory and anti-fibrotic effects [38]. In two large phase 3 studies in patients with DKD (FIDELIO- and FIGARO-DKD), finerenone was significantly more effective than standard of care including ACEi and ARBs in slowing the decline in estimated glomerular filtration rate (eGFR) and improving cardiovascular outcomes, with non-significant reductions in end-stage kidney disease and all-cause mortality [38, 97].

**Table 3:** Recent and ongoing clinical trials of anti-inflammatory drugs in patients with DKD

Drug (target)	Study design	Intervention and comparison	Model compartment (Fig. 2)	Primary efficacy outcome	Remarks
Bardoxolone (Nrf2 activator)^a^ [127, 128]	Phase 3, randomized,double-blind trial (BEACON) in adults with T2DM and eGFR of 15 to < 30 mL/min/1.73 m^2^	Bardoxolone 20 mg/day or placebo plus background conventional therapy	Filtration Resorption	No effect on rate of ESRD or death from cardiovascular causes (HR, 0.98; 95% CI: 0.70, 1.37; *P* = 0.92)	Terminated because of higher rate of cardiovascular events than placebo, but GFR improved vs placebo
Bardoxolone (Nrf2 activator)^a^ [109]	Phase 2, randomized, double-blind, placebo-controlled study (TSUBAKI) in adults with T2DM, CKD stage 3–4, and UACR < 300 or < 2000 mg/g	Bardoxolone 15 mg/day or placebo for 16 weeks plus ACEi and/or ARB	Filtration Resorption	Improved GFR from baseline to week 16 (mean, 5.95 [95% CI: 2.29, 9.60] vs –0.69 [–3.83, 2.45)] mL/min/1.73 m^2^; *P* = 0.008)	Improved GFR; no safety signals of concern detected
Finerenone (mineralocorticoid receptor antagonist)^b^ [38]	Phase 3, randomized, double-blind, placebo-controlled study (FIDELIO-DKD) in adults with T2DM and CKD receiving ACEi or ARB	Finerenone 10 or 20 mg/day or placebo plus guideline-directed therapy	Filtration Resorption	Reduced risk of kidney failure, sustained eGFR decrease or death from renal causes (HR, 0.82; 95% CI: 0.73, 0.93; *P* = 0.001)	Discontinuation due to hyperkalemia in 2.3% of patients receiving finerenone
Selonsertib (ASK1 inhibitor) [129]	Phase 2, randomized, placebo-controlled study in adults with T2DM and treatment-refractory moderate-to-advanced DKD	Selonsertib 2, 6, or 18 mg/day or placebo	Filtration Resorption Immune cell recruitment	No improvement in eGFR from baseline to week 48. Week 4 to 48 post hoc difference vs placebo: 3.11 mL/min/1.73 m^2^/year (95% CI: 0.10, 6.13; nominal *P* = 0.043)	Acute inhibitory effects on creatinine secretion confounded eGFR differences from baseline
Baricitinib (JAK1/JAK2 inhibitor) [94]	Phase 2, randomized, double-blind, placebo-controlled study in adults with T2DM, eGFR or 25–70 mL/min/1.73 m^2^, UACR of 300–5000 mg/g on ACEi or ARB	Baricitinib 0.75, 1.5 or 4 mg/day or 0.75 mg twice daily or placebo	Immune cell recruitment	Improvement in UACR at week 24 at highest dose (ratio to baseline, 0.59; 95% CI: 0.38, 0.93; *P* = 0.022), but effects not dose-dependent	Increased risk of anaemia. Terminated for business reasons
MEDI3506 (IL-33 mAb) NCT04170543	Phase 2b, randomized, double-blind, placebo-controlled study in patients with DKD and eGFR of 30–75 mL/min/1.73 m^2^ on ACEi or ARB	MEDI3506 or placebo for 24 weeks, plus dapagliflozin in weeks 12–24	Filtration Resorption Immune cell recruitment	Change in UACR from baseline to week 24	Recruiting
AZD5718 (FLAP inhibitor) NCT04492722	Phase 2b, randomized, double-blind, placebo-controlled study in patients with eGFR of 20–75 mL/min/1.73 m^2^ and UACR of 200–5000 mg/g (DKD in a subgroup)	AZD5718 or placebo for 20 weeks, plus dapagliflozin in weeks 12–20	Immune cell recruitment Filtration Resorption	Change in UACR from baseline to week 20	Recruiting
ASP8232 (VAP1 inhibitor) [110]	Phase 2, randomized, double-blind, placebo-controlled study in adults with T2DM, CKD, UACR of 200–3000 mg/g, eGFR of 25–75 mL/min/1.73 m^2^, HbA1c of < 11·0% (< 97 mmol/mol) on ACEi or ARB and anti-diabetic medication	ASP8232 40 mg/day or placebo for 12 weeks	Immune cell recruitment	Improvement in UACR at week 12 (difference versus placebo, –19.5% 95% CI: –34.0, –1.8; *P* = 0·033)	Increased risk of peripheral oedema and anaemia. Terminated for business reasons
PF-04634817 (CCR2 and CCR5 receptor dual antagonist) [130]	Phase 2 randomized, double-blind, placebo-controlled study in patients with T2DM, eGFR of 20–75 mL/min/1.73 m^2^ and UACR ≥ 30 mg/g	PF-04634817 150 or 200 mg/day (depending on eGFR) or placebo	Immune cell recruitment	Placebo-adjusted improvement in UACR of 8.2% (ratio 0.918; 95% credible interval: 0.75, 1.09) at week 12	Clinical development halted owing to insufficient efficacy
Propagermanium / DMX-200 (CCR2 inhibitor) [131]	Randomized, open-label, pilot trial in patients with T2DM, dipstick proteinuria ≥ 1 + or UACR of ≥ 30 mg/g and eGFR of ≥ 30 mL/min/1.73 m^2^	Propagermanium 30 mg/day for 12 months plus usual care or usual care alone	Immune cell recruitment	No change in UACR from baseline to 12 months (change, 25.0%; 95% CI: − 20.4, 96.5; *P* = 0.33)	Ineffective
Propagermanium / DMX-200 (CCR2 inhibitor) NCT03627715 [132]	Phase 2 randomized, double-blind, placebo-controlled crossover trial in patients with DKD already on irbesartan 30 mg/day and an eGFR of 25–90 mL/min/1.73 m^2^ and UACR of 30–500 mg/mmol	Propagermanium twice daily or placebo for 12 weeks	Immune cell recruitment	22% placebo-adjusted reduction in albuminuria from baseline (not powered for inferential statistical analysis)	Positive efficacy data announced in press release
CCX140-B (CCR2 inhibitor) [133]	Phase 2 randomized, double-blind, placebo-controlled trial in patients with T2DM, proteinuria and eGFR ≥ 25 mL/min/1.73 m^2^ on anti-diabetic medication and ACEi or ARBs	CCX140-B 5 mg/day or 10 mg/day or placebo for 12 weeks (amended to 52 weeks)	Immune cell recruitment	Improvement in UACR from baseline to week 52 (placebo-adjusted difference of –16% for 5 mg [one-sided upper 95% CI –5%; *P* = 0.01] and –10% for 10 mg [+ 2%; P*p* = 0.08])	Authors concluded potential renoprotective effects, but these were not dose-dependent. No further studies in patients with DKD
Bindarit (NF-κB modulator) [134] NCT01109212	Phase 2, randomized, double-blind, placebo-controlled study in patients with DKD receiving irbesartan	Bindarit 600 mg twice daily or placebo plus irbesartan 300 mg/day for 12 weeks	Immune cell recruitment	Change in urinary albumin excretion (µg/mL) from baseline	Reduced albuminuria reported in congress abstract, but full results not published and no further studies
Gevokizumab (IL-1β mAb) 2013–003,610-41	Phase 2, randomized, double-blind, placebo-controlled study in patients with DKD and eGFR of 20–60 mL/min/1.73 m^2^ and UACR > 300 mg/g	Gevokizumab 3, 10, 30 or 60 mg or placebo for 52 weeks	Immune cell recruitment	Change in measured GFR from baseline	Terminated for ‘strategic reasons unrelated to safety’
Canakinumab (IL-1β mAb) [135]	Subgroup analysis of phase 3 trial (CANTOS) in patients who were stable after myocardial infarction with hsCRP ≥ 2 mg/mL and eGFR < 60 mL/min/1.73m^2^	Canakinumab 50, 150 or 300 mg or placebo	Immune cell recruitment	Reduced risk of major adverse cardiovascular events (HR, 0.82; 95% CI: 0.53, 0.86; *P* = 0.0015)	No clinically meaningful improvement or worsening of eGFR or UACR or renal AEs
Emapticap pegol (CCL2 binding aptamer) [136]	Phase 2, randomized, double-blind, placebo-controlled study in patients with eGFR > 25 mL/min/1.73 m^2^ and UACR > 100 mg/g	Emapticap 0.5 mg/kg twice weekly or placebo for 12 weeks	Immune cell recruitment	No significant improvement in UACR from baseline to week 12 or to 8 weeks after discontinuation	Suggestion of efficacy in a post hoc analysis excluding some patients, but no further studies

^a^Anti-oxidant or anti-inflammatory mechanism of actions unclear

^b^Natriuretic and anti-inflammatory mechanisms of action

Abbreviations: *ACEi* angiotensin-converting enzyme inhibitor, *AE* adverse event, *ARB* angiotensin receptor blocker, *ASK1* apoptosis signal-regulating kinase 1 (mitogen-activated protein kinase kinase kinase 5), *CCL2* C–C motif ligand 2, *CCR2* C–C chemokine receptor type 2, *CCR5* C–C chemokine receptor type 5, *CI* confidence interval, *CKD* chronic kidney disease, *DKD* diabetic kidney disease, *Egfr* estimated glomerular filtration rate, *FLAP* 5-lipoxygenase-activating protein, *GFR* glomerular filtration rate, *HbA1c* glycated haemoglobin, *hsCRP* high-sensitivity C-reactive protein, *IL-1β* interleukin-1β. *IL-33* interleukin-33, *JAK* Janus kinase, *mAb* monoclonal antibody, *T2DM* type 2 diabetes mellitus, *UACR* urine albumin-to-creatinine ratio, *VAP1* vascular adhesion protein 1

A key challenge for the development of new anti-inflammatory medicines is the limited understanding of relevant surrogate endpoints for early clinical development before phase 3 [98] Most published trials have used the traditional markers of UACR and eGFR both as baseline patient selection criteria and as efficacy measures (Table 3). So far, the most reliable surrogate kidney endpoints seem to be a 30% improvement in albuminuria within 6 months, time to 30% decline in eGFR from baseline, and mean reduction in the slope of eGFR decline greater than 0.5–1.0 mL/min/1.73 m^2^/year over at least 2 years [99–102]. A sample size of approximately 100 patients per arm provides 80% power to detect a 30% reduction in UACR at 6 months, making it an appealing surrogate endpoint for a phase 2 efficacy study [103]. However, short-term UACR parameters have a legacy from studies of anti-hypertensive medications which may not be appropriate for studies of anti-inflammatory drugs. Rates of progression in UACR vary substantially from patient to patient due to differences in underlying pathophysiology, as well as race, comorbidities and sex [104, 105]. The resulting inflammation-independent diversity presents a challenge to adequate statistical powering of clinical studies, even assuming that the investigational drug can improve short-term UACR in ‘all comers’ with DKD. Also, albuminuria-based endpoints do not differentiate between glomerular and tubular loss of protein (the ‘filtration’ and ‘resorption’ compartments of our conceptual model). In the future, the over-all prevalence of albuminuria in patients may, furthermore, decline substantially with increasing take-up of standard care involving anti-hypertensive medications and SGLT2 inhibitors [106–108].

Lack of efficacy on renal functional outcomes like UACR or eGFR led to failure of several drug classes at phase 2, including those targeting pathways implicated in the pathophysiology of DKD (e.g. IL-1β antibodies and Janus kinase inhibitors). Unpredictable adverse drug reactions led to failure for bardoxolone (activator of the Nrf2 pathway and an inhibitor of the NF-κB pathway) and the vascular adhesion protein 1 inhibitor ASP8232, despite promising phase 2 kidney function outcomes (Table 3) [109, 110]. The generally disappointing results of trials of anti-inflammatory therapies to date hence indicate a need for improved, compartment-focused selection criteria and outcome measures.

Ziltivekimab, a fully human monoclonal antibody directed against the IL-6 ligand, was evaluated in a randomised, double-blind, phase 2 trial involving individuals (*n *= 264) with elevated high-sensitivity CRP and chronic kidney disease [111]. The primary study outcome was percentage change from baseline in high-sensitivity CRP after 12 weeks of treatment with ziltivekimab (7.5 mg, 15 mg and 30 mg) compared with placebo. Biomarker and safety data were collected over 24 weeks of treatment. After 12 weeks, median high-sensitivity CRP levels were reduced by 77% for the 7·5 mg group, 88% for the 15 mg group, and 92% for the 30 mg group compared with 4% for the placebo group. Dose-dependent reductions in fibrinogen, serum amyloid A, haptoglobin, secretory phospholipase A2, and lipoprotein(a) were observed. Ziltivekimab was well tolerated. Based on these data showing markedly reduced biomarkers of inflammation and thrombosis relevant to atherosclerosis a further trial is planned to investigate the effect of ziltivekimab in patients with chronic kidney disease, increased high-sensitivity CRP, and established cardiovascular disease.

### Patient selection based on predominant immunopathology

Maximizing the potential benefits of new treatments involves identifying the compartments most affected by immunopathology in individual patients with DKD. The five-compartment model may serve as a guide for development of tools and therapies that will enable physicians to provide the right treatment to the right patients consistently and accurately, ideally without the need for kidney biopsy. Approaches that will allow patient classification include genomic and transcriptomic studies and identification of novel fluid-phase and imaging biomarkers. However, robust interventional trials are still needed to fully validate these exploratory endpoints.

Circulating biomarkers may allow patients to be identified based on molecular features of inflammation, and stratified based on predominant immunopathology [112]. For example, plasma levels of TNFR-1, TNFR-2 and kidney injury molecule 1 (KIM-1) are associated with decline in eGFR, even after adjustment for baseline albuminuria and eGFR, in multiple cohorts of patients with type 2 diabetes [5, 35, 87, 89, 90, 113]. A proteomics study recently identified a ‘kidney risk inflammatory signature’ comprising a cluster of 17 circulating inflammatory biomarkers that strongly associate with development of end-stage renal disease in multiple ethnicities [34]. Although the cluster included pro-inflammatory mediators already implicated in DKD (e.g. CCL2), it also included chemokines and ILs that were not known to be associated with the disease [34]. This suggests that relevant immunopathological pathways remain to be elucidated.

Inflammatory responses stimulated by toll-like receptors (TLRs), notably TLR4, appear to play a decisive role in the progression of DKD [114, 115] while complement dysregulation, may also contribute to progression [116]. Furthermore, the renal NF-κB pathway, implicated in the development and progression of experimental DKD, may also become an important therapeutic target [117].

Genomic and transcriptomic studies also offer routes to discovery of novel markers. Academic-industry systems biology consortiums aim to share molecular target identification efforts and expertise to accelerate novel drug development (e.g. the Renal Pre-Competitive Consortium [RPC^2^]) [118]. In a recent RNA-seq study, micro-dissected glomerular and tubulointerstitial kidney biopsy tissue fractions were analyzed from patients with DKD and matched living kidney donors. The results confirmed inflammatory responses, complement activation and extracellular matrix deposition as key pathophysiological processes [119]. A whole-exome sequencing study, in 3315 patients with chronic kidney disease and 9536 controls, used in vivo and in vitro approaches to validate the most strongly associated genes as potential novel diagnostic or therapeutic targets [120]. The analysis identified 93 genes with a strong CKD correlation which after ranking based on literature data supporting a link to CKD relevant biology resulted in 31 genes that were further evaluated in vitro and in vivo. Ultimately a single gene was identified as a CKD target that entered the pipeline for drug discovery.

Magnetic resonance imaging (MRI) can detect changes that precede albuminuria and GFR decline in patients with DKD [121]. Ischaemic regions of the kidney can be identified using blood oxygenation level-dependent MRI, and these signals are predictive of chronic kidney disease progression [122, 123]. Renal fibrosis identified using diffusion-weighted MRI may detect DKD progression earlier than eGFR [124]. Early impairment in renal perfusion in patients with diabetes can be identified using arterial spin labelling MRI, and these signals correlate with reduced GFR [125].

Combining these rapid advances in histology, genetics, ‘omics’, and imaging may unlock the potential of anti-inflammatory therapies in DKD. Eventually, patient stratification by specific and relevant pathophysiology, integrated with pre-clinical models of these disease processes, may allow intervention with novel targeted therapies in the right patients at the right time.

Nevertheless, limitations of the five-compartment model include that not all patients with histopathological indicators of DKD will ultimately develop the condition [126].

## Conclusion

Compelling evidence indicates that sub-clinical chronic inflammation plays a key role in the development and progression of DKD. Successful development of novel anti-inflammatory therapies will involve targeting specific pathways in specific patients with DKD. Novel medicines will not be unique to each individual but will be tailored for optimal treatment of particular subgroups of the patient population. This precision medicine approach has the potential to maximize positive health outcomes while minimizing unnecessary side effects and costs, but it requires a significantly improved understanding of DKD. Our conceptual model provides a framework for identifying and assessing novel drugs that act in five key compartments of kidney function: immune cell recruitment and activation; filtration; resorption and secretion; extracellular matrix regulation; and perfusion. The model is intended to inform selection of pre-clinical models to identify and validate candidates for clinical testing, as well as design of clinical trials with selection criteria and efficacy measures that can provide early evidence of clinical benefit in patients with DKD. The aim of the model is to prevent cardiovascular mortality and progression to end-stage renal disease, which will remain high in patients with type 2 diabetes and chronic kidney disease despite recent improvements to standard of care.

## Data Availability

Not applicable.
